# Patient Satisfaction Following Phlebectomy for Prominent Temporal and Periorbital Facial Veins—Results From Our First Cohort of Patients

**DOI:** 10.1111/jocd.70283

**Published:** 2025-06-06

**Authors:** Simon D. Muschamp, Leonardo Da Silva, Amanda G. Nielsen, Mark S. Whiteley

**Affiliations:** ^1^ The Whiteley Clinic Surrey UK

**Keywords:** facial veins, periorbital veins, phlebectomy

## Abstract

**Background:**

Prominent veins in the temporal and periorbital regions of the face are a significant cosmetic concern, impacting self‐confidence and often prompting patients to seek treatment. While transcutaneous laser and sclerotherapy are effective for treating flat or superficial veins, bulging veins frequently require surgical removal via phlebectomy. Despite growing interest in facial vein treatments, there is limited published research evaluating patient satisfaction following facial phlebectomy. This study aimed to assess patient satisfaction in our first cohort of patients.

**Methods:**

A retrospective review was conducted on 22 patients who underwent facial phlebectomy, primarily for temporal and periorbital veins, between June 2015 and October 2021. The procedure was performed under local anesthesia using micro‐incisions and hook phlebectomy. Post‐treatment outcomes were assessed using a structured questionnaire, collecting qualitative feedback and quantitative data via a 5‐point Likert scale (1 = worst, 5 = best).

**Results:**

Of the 22 patients, 13 responded to the questionnaire (59% response rate), with 12 providing usable data. Respondents' mean age was 45.4 years (range 28–64), with 10 females and 2 males. Mean satisfaction scores were: (1) Vein disappearance: 3.8/5. (2) Scar appearance: 4.4/5. (3) Overall satisfaction: 4.2/5. (4) Willingness to recommend: 4.1/5.

No complications such as infection, numbness, persistent pain, or significant scarring were reported.

**Conclusion:**

Phlebectomy for temporal and periorbital veins is a safe and effective procedure for larger facial veins unsuitable for transcutaneous laser treatment. High satisfaction rates and minimal complications support its continued use as a cosmetic treatment option. Further research involving larger cohorts, objective assessments, and fixed follow‐up intervals is recommended to validate these findings and optimize patient outcomes.

## 1 Introduction

Visible facial veins in the temporal and periorbital regions are a common cosmetic concern, often prompting individuals to seek treatment to improve their self‐confidence. These veins may appear flat and green or blue, or present as bulging. Treatment options include transcutaneous laser [1], sclerotherapy [2], electrosurgery [3], and surgical phlebectomy [4].

Among these transcutaneous lasers, the Nd:YAG laser is one of the most frequently used methods for superficial facial veins. It works by targeting blood within the vessel to generate heat and induce venous closure [5]. However, its efficacy declines with larger veins (> 3–4 mm), which often require surgical intervention [6, 7].

Facial phlebectomy is a minimally invasive procedure that removes the vein through a small incision using a phlebectomy hook. While aesthetic phlebology is an area of growing interest, there is limited published research evaluating outcomes, particularly patient satisfaction, following facial phlebectomy.

This study aimed to evaluate patient satisfaction in our first cohort of facial phlebectomy patients as a key measure of treatment efficacy. These findings may help guide future treatment decisions and inform patient expectations.

## 2 Patients and Methods

**FIGURE 1:**
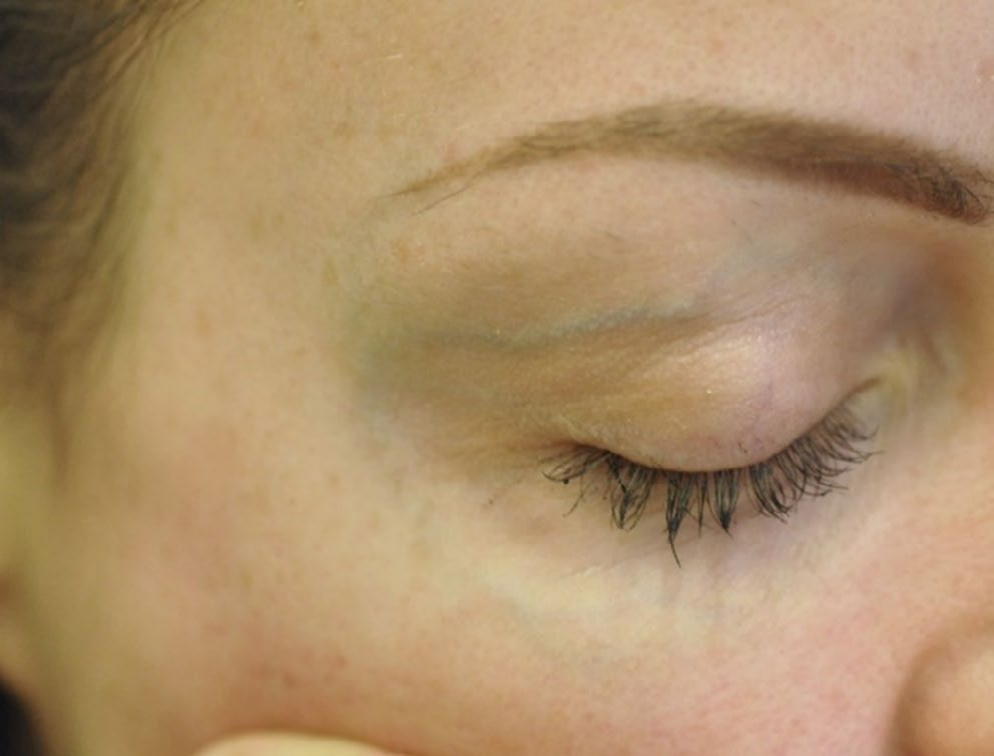
Pre‐operative image before facial phlebectomy.

Since 2015, we have offered surgical treatment to patients with large or prominent temporal or periorbital facial veins who had either failed or were deemed unsuitable for transcutaneous Nd:YAG (1064 nm) laser treatment (Figure 1).

Following assessment by the senior author (MSW), suitable patients underwent a consenting process during which the limited experience and literature regarding facial phlebectomy were explained.

A total of 22 patients were treated between June 2015 and October 2021. Patients were later contacted by e‐mail and, where necessary, by telephone to assess post‐treatment outcomes via questionnaire.

### 2.1 Surgical Technique

The current standard procedure is outlined below.

**FIGURE 2:**
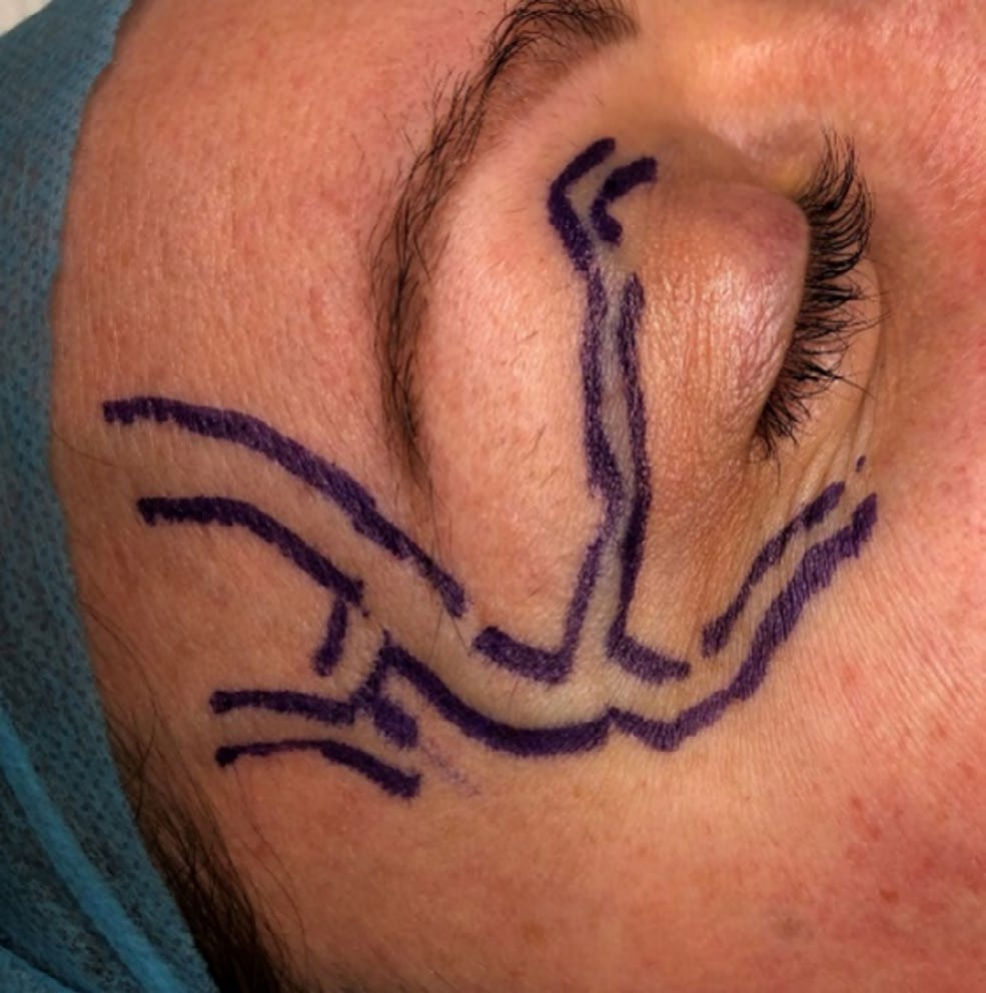
Marking of facial veins prior to phlebectomy.

Patients were positioned with the target vein uppermost. The operating room was kept warm to promote venous dilation, and the table was tilted approximately 10° head‐down (Trendelenburg) to allow the veins to fill. Veins were marked using a permanent skin marker (Figure 2).

The table was then returned to horizontal, and the patient was handed a mirror to confirm vein markings. The patient was re‐positioned and the table tilted 10° head up (reverse Trendelenburg) to empty the veins.

**FIGURE 3:**
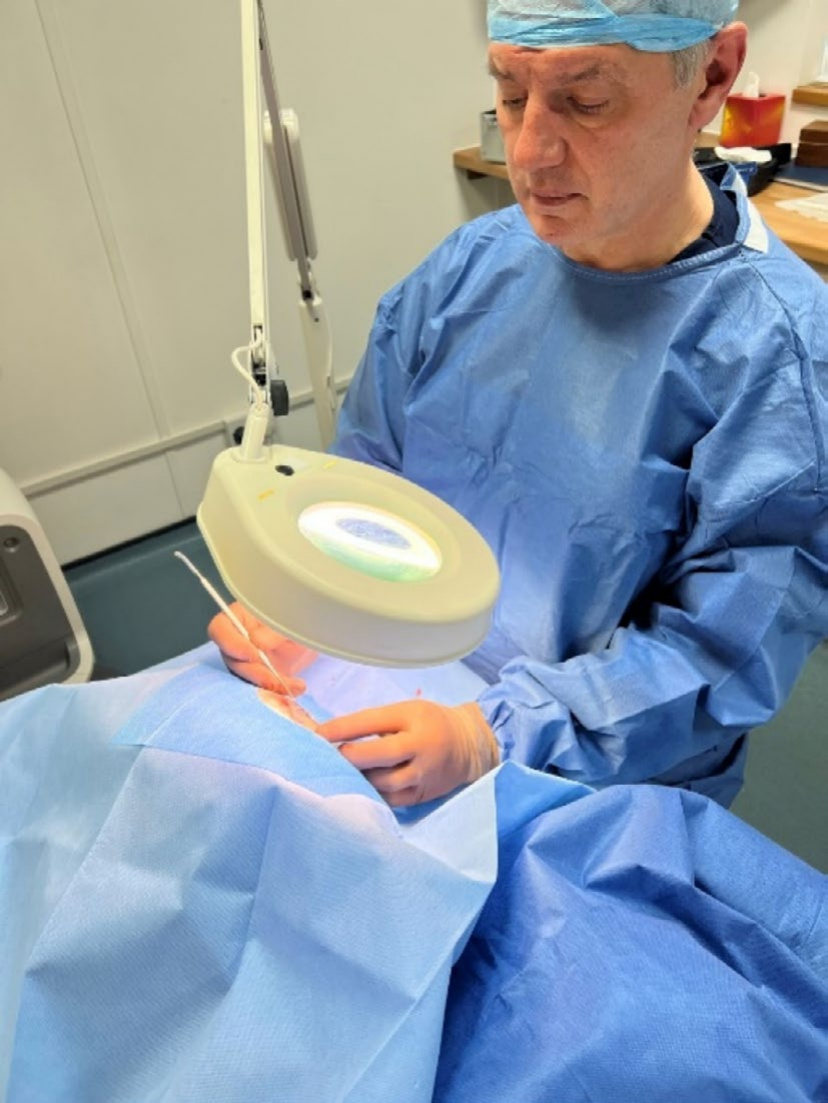
Initial illumination and magnification in phlebectomy surgery.

**FIGURE 4:**
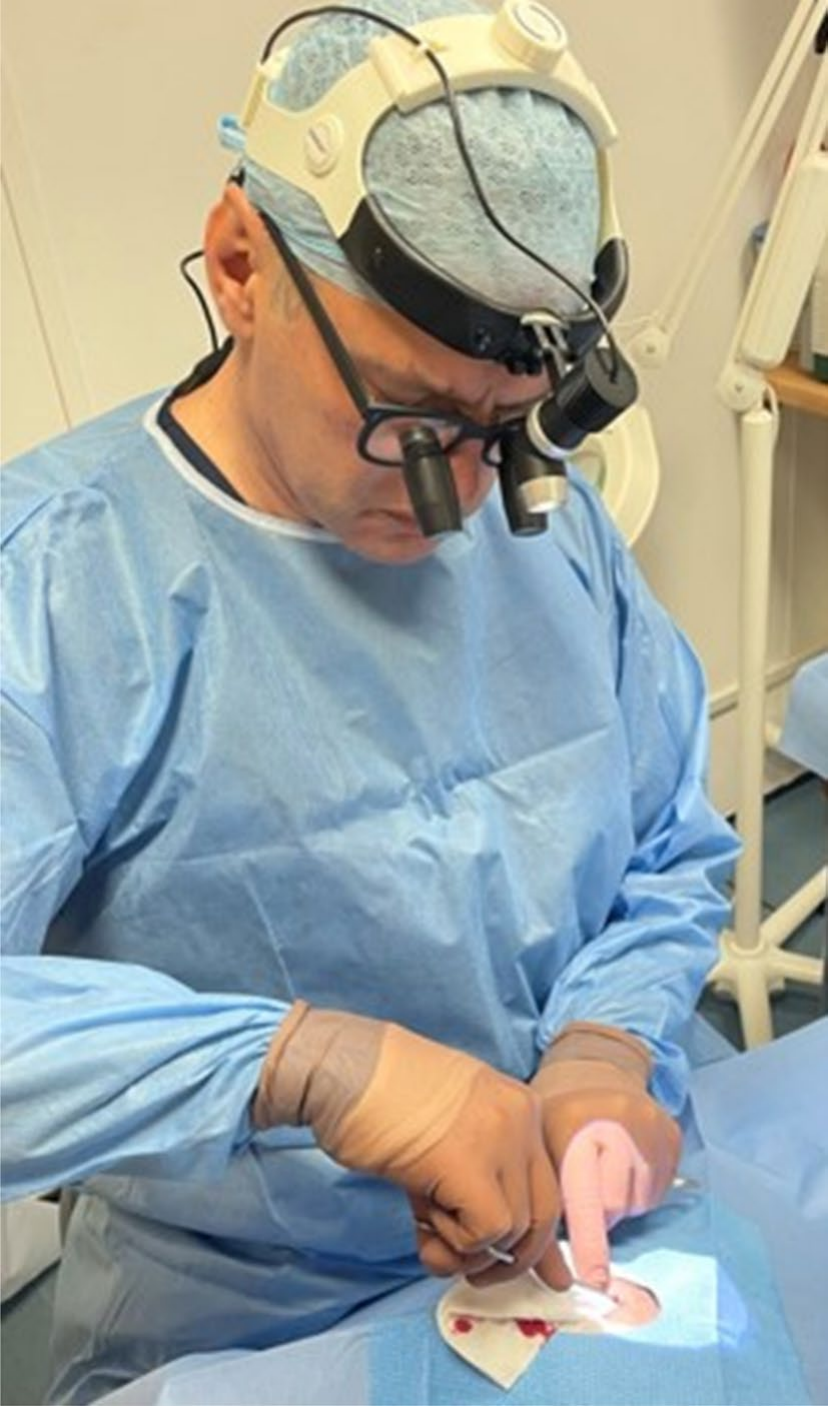
Surgical loupes and LED headlamp for improved vein removal.

Early cases used a freestanding magnifying lamp (Figure 3), while later cases employed 7× surgical loupes (CLS Med Ltd., Sheffield, UK) and an LED headlight (Daray Medical Lighting, Swadlincote, UK) for improved visualization (Figure 4).

The skin was cleansed with antiseptic and draped. Local anesthesia (2% lignocaine with epinephrine; Torbay Pharma, Paignton, UK) was administered using a 30G yellow hypodermic needle (BD Microlance, Becton Dickinson SA, Spain). Incisions were made directly over the vein, aligned with skin creases, using a “beaver” fine surgical blade (Swann‐Morton, Sheffield, UK). Each incision was made at an oblique angle to create a skin flap, which was later closed with cyanoacrylate glue.

**FIGURE 5:**
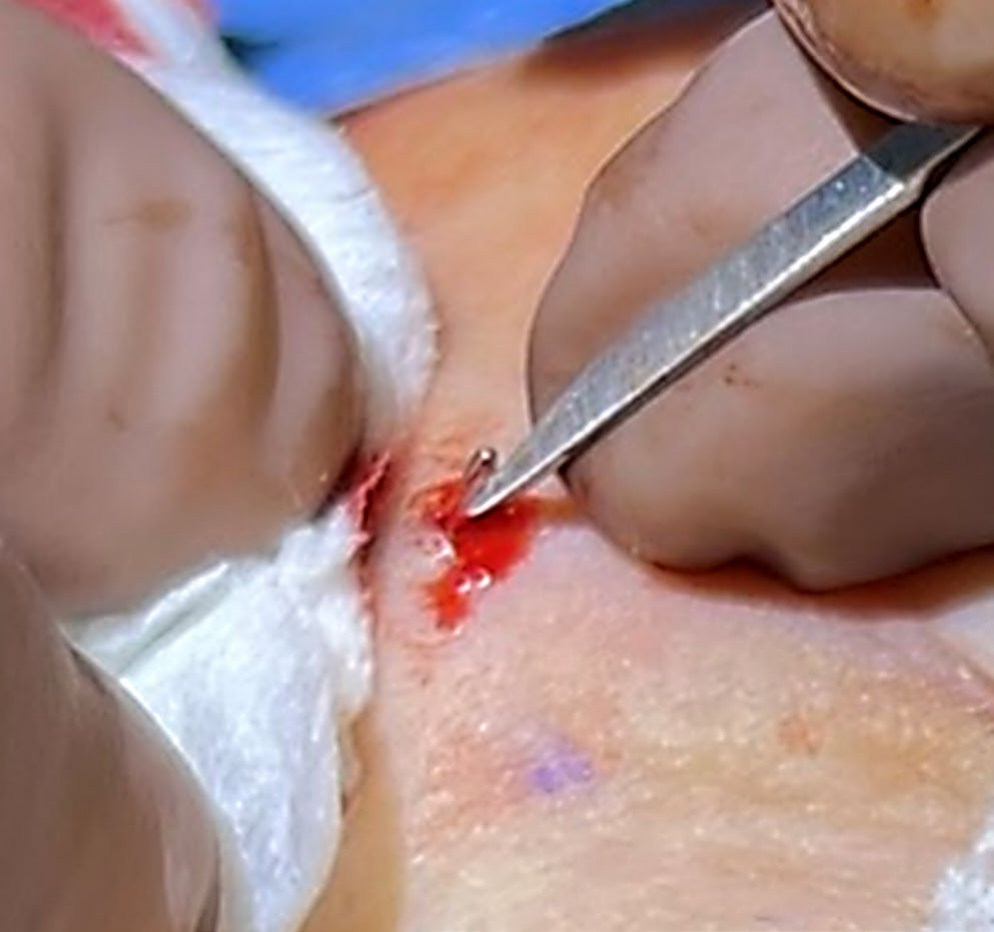
Surgical extraction of the vein using Phlebectomy hook.

A disposable Varaday‐style phlebectomy hook (Blink Medical, part of Corza Medical, Solihull, UK) was inserted to extract the vein (Figure 5). Facial veins were more challenging to access than leg veins due to the superficial musculoaponeurotic system (SMAS) [8].

**FIGURE 6:**
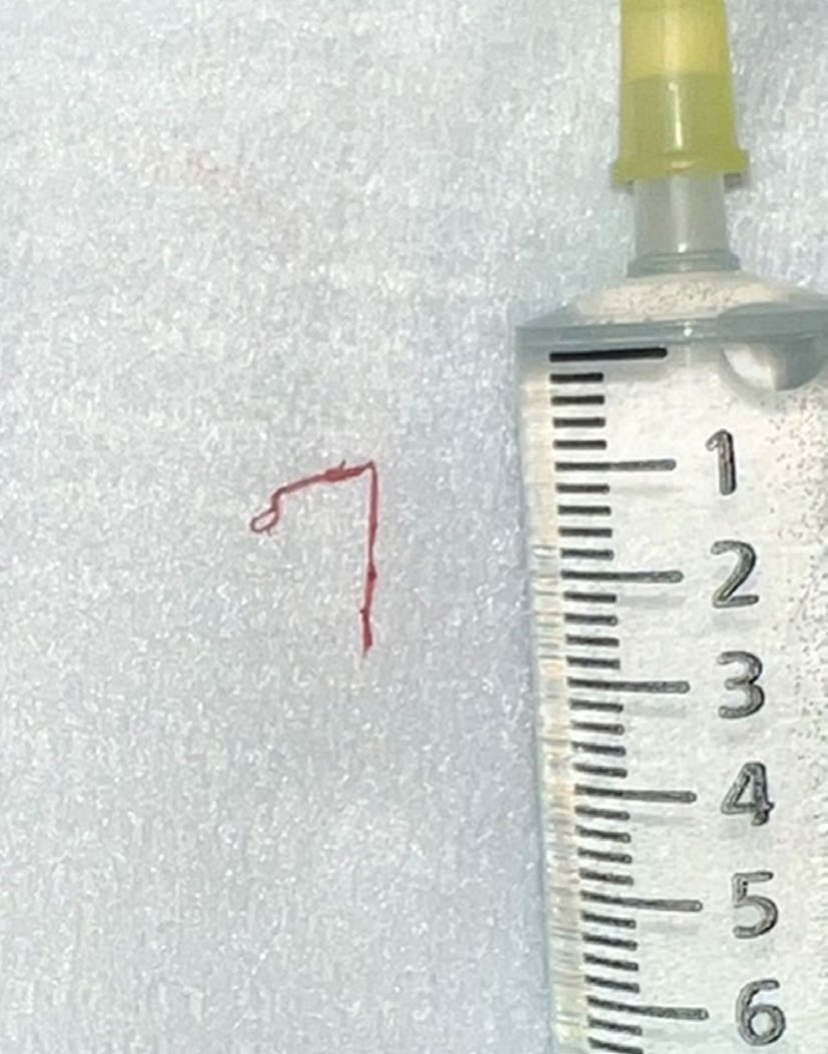
Post‐phlebectomy vein segment in comparison with 10 mL syringe.

Veins were grasped with disposable neurosurgical needle holders (DTR Medical, Swansea, UK), as standard haemostatic clips were ineffective at this scale (Figure 6). Veins were removed via gentle traction and agitation.

**FIGURE 7:**
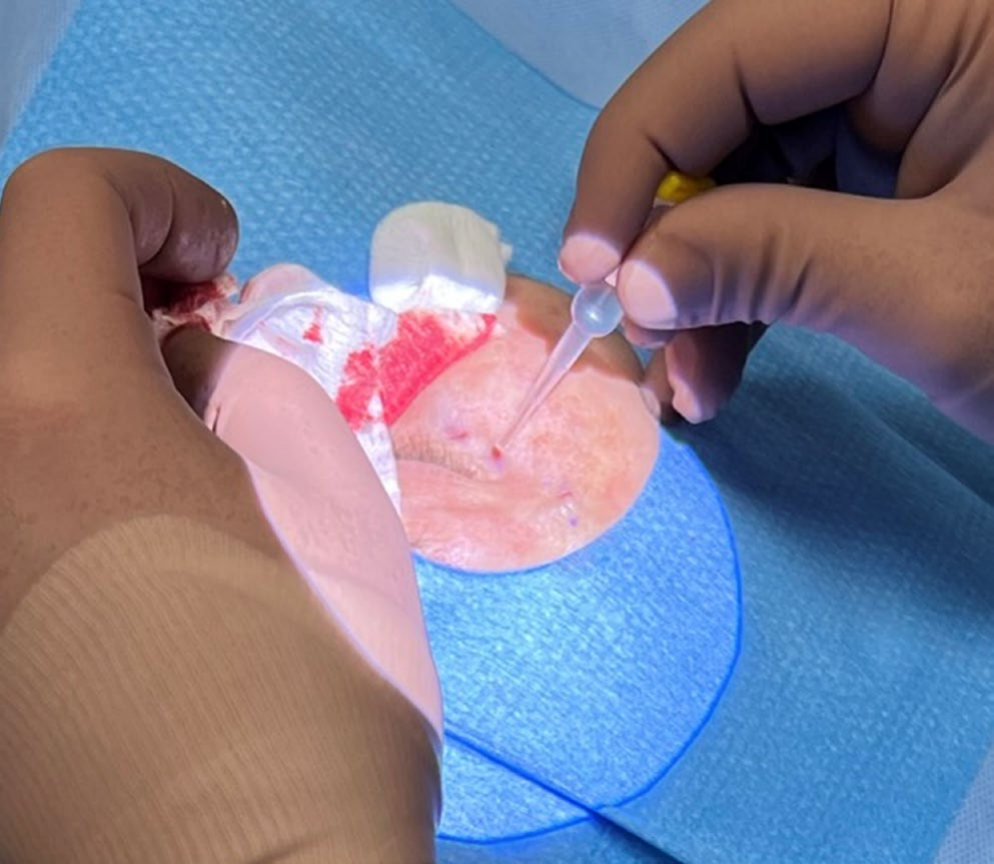
Wound closure using colorless cyanoacrylate glue.

Minor bleeding was controlled with digital pressure or the vasoconstrictive effects of epinephrine. The skin was closed by replacing the upper flap and sealing it with colorless cyanoacrylate glue (Histoacryl, B Braun Surgical, Barcelona, Spain) (Figure 7).

**FIGURE 8:**
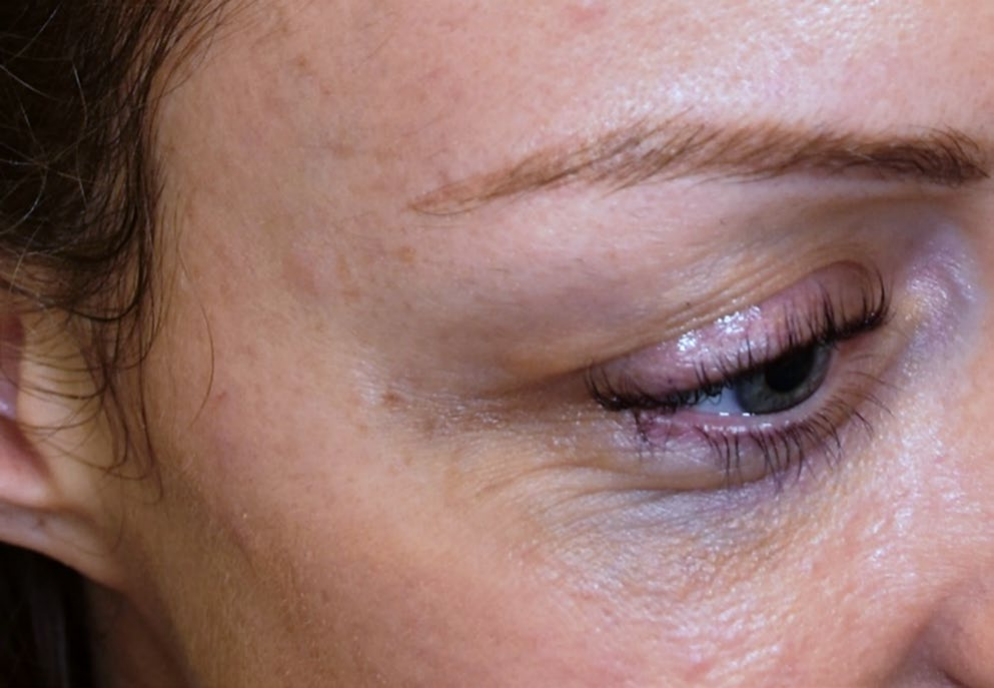
Post‐operative image after facial phlebectomy.

A post‐operative image taken 8 weeks later showed minimal scarring and a well‐healed appearance (Figure 8). In addition to patient‐reported outcomes, all patients were reviewed approximately 4–6 weeks post‐procedure by the treating vascular surgeon. At this follow‐up, the treated area was assessed in person for cosmetic and procedural outcomes, and any concerns or residual veins were discussed. Although not scored formally, these reviews were documented contemporaneously in clinical notes and served as a routine form of objective assessment.

### 2.2 Questionnaire

A retrospective review identified 22 patients treated between June 2015 and October 2021. These individuals were contacted by email and telephone to assess their surgical outcomes.

**FIGURE 9:**
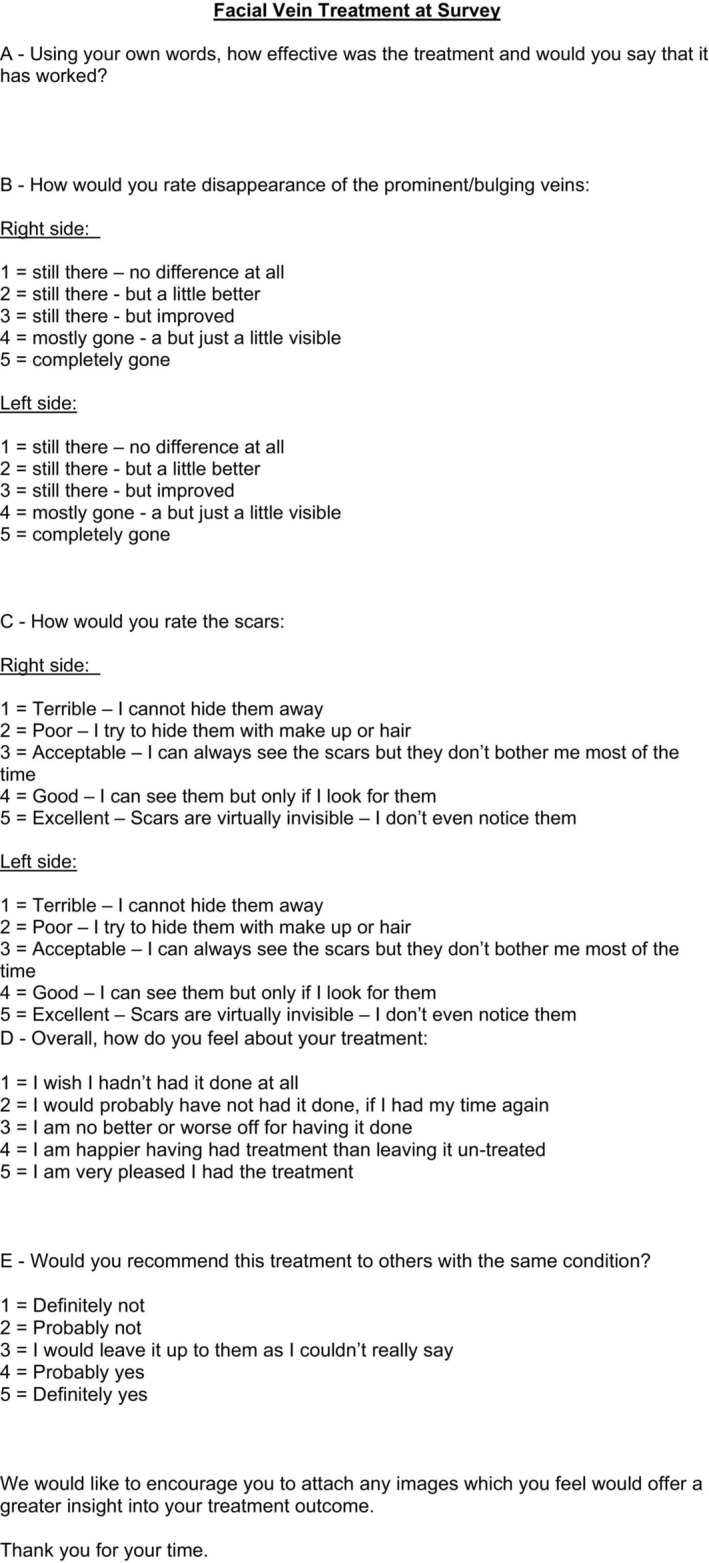
Questionnaire template for patient feedback on treatment outcomes.

The questionnaire gathered both qualitative and quantitative feedback (Figure 9). The first section addressed perceived treatment efficacy, while the second used a 5‐point Likert scale to evaluate vein disappearance, scar prominence, overall satisfaction, and willingness to recommend the procedure. Patients were also encouraged to submit photographs documenting post‐treatment outcomes.

This study did not require ethics approval according to the HRA decision tools (https://www.hra‐decisiontools.org.uk/ethics/).

## 3 Results

Despite multiple contact attempts, a response rate of 59% was achieved, with 13 participants responding. One questionnaire file was corrupted, leaving 12 usable responses. Among these, 10 were female and 2 were male, with a mean age of 53.0 years (range 38–65). To assess potential selection bias, responders were compared with non‐responders. The mean age of non‐responders was 47.5 years (range 30–62), and 69% were female. No substantial demographic differences were identified between groups.

**FIGURE 10:**
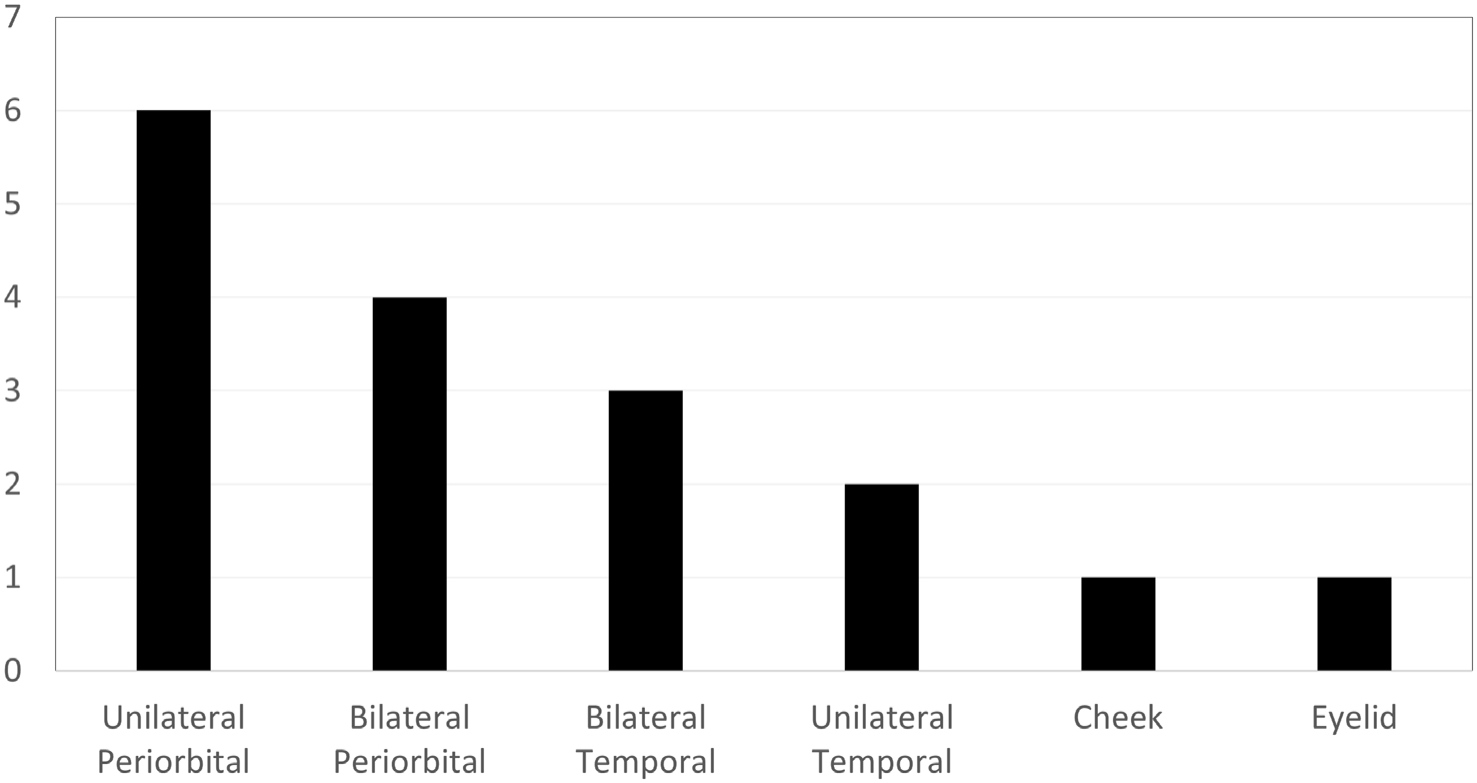
Frequency and position of treated facial veins.

The frequencies and locations of treated veins are summarized in Table 1 and Figure 10, with periorbital veins being the most commonly treated, followed by temporal veins. Cheek and eyelid veins were treated less frequently and are not the primary focus of this study.

**TABLE 1:** Time of patient responses since treatment.

Time since procedure	Frequency	%
< 1 year	2	17%
1–2 years	0	0%
2–3 years	2	17%
3–4 years	1	8%
4–5 years	6	50%
5–6 years	0	0%
6–7 years	1	8%

As this was a retrospective study, response timing varied. Most responses (50%) were received 4–5 years post‐treatment, and only 17% occurred within the first year. These data are presented in Table 1.

Patients first provided open‐ended feedback (Table 2), followed by quantitative satisfaction scores (Table 3).

**TABLE 2:** Qualitative patient feedback on treatment effectiveness and experience.

“My facial veins were fully removed with no pain or scarring”
“The treatment was very effective”
“The treatment received was very effective and has made a massive difference to me”
“I would say that the treatment has worked well and has been approximately 90% effective”
“The treatment hasn't worked out as well as I'd hoped. The removal of the veins on my face went fine. I was still left with some veins on my eyelid, which I was told could happen, but I was disappointed they didn't come out well as they are very visible”
“The bulging veins have gone… fine ones have regrown”
“I had a large unsightly bulging vein removed successfully, but the vein re‐appeared further down and over my eyelid”
“Very effective. More painful than I thought, however, glad I had it done”
“The treatment was extremely effective, and I would say that it has worked perfectly. The veins have gone and there are not even visible scars”
“Yes it has worked in that the veins treated have gone, however they have been replaced by new veins in the area which are now prominent”
“The treatment has not been that effective”
“It was very straight forward, fast and almost pain free. This procedure did not remove all the veins but I can see it is better than before”

**TABLE 3:** Quantitative patient satisfaction scores following facial phlebectomy.

Question	Mean	Range
How would you rate the disappearance of the prominent/bulging veins?	3.8	2–5
How would you rate the scars?	4.4	3–5
Overall, how do you feel about your treatment?	4.2	2–5
Would you recommend this treatment to others with the same condition?	4.1	2–5

While most patients reported successful vein removal with minimal scarring, some noted mild recurrence or the development of new veins. Experiences with pain varied, and a small number expressed dissatisfaction due to persistent veins in challenging areas such as the eyelids.

Quantitative data supported these observations, with a mean overall satisfaction score of 4.2 out of 5.

Scar appearance received the highest rating, followed by overall satisfaction and willingness to recommend the procedure. Vein disappearance received the lowest rating, reflecting occasional recurrence or residual veins.

These findings support facial phlebectomy as a procedure associated with high patient satisfaction, especially in cosmetic outcomes, despite some limitations such as recurrence or regrowth of fine veins.

## 4 Discussion

Interest in aesthetic phlebology is growing, particularly in the treatment of facial veins. Historically, treatments have focused on thread veins near the nose and cheeks, while larger facial veins were often considered untreatable [9].

The 1064 nm Nd:YAG laser is commonly used for facial reticular veins and is reported to have patient satisfaction rates up to 79% [10]. However, its effectiveness diminishes with veins over 3 mm in diameter [11] and it carries risks such as burns, erythema, and hyperpigmentation which are undesirable in cosmetic settings.

Sclerotherapy is another treatment option [12], but its use in the periorbital and temporal regions carries significant risk due to anatomical proximity to critical vascular structures. Reported complications include ulceration [13], orbital pain [14], and rare cases of blindness [15]. For this reason, we do not recommend sclerotherapy in these areas.

Literature on facial phlebectomy reports high efficacy and minimal scarring up to 5 years post‐procedure [4, 16], though few studies evaluate patient satisfaction. Our results support existing data, confirming that phlebectomy is minimally invasive, effective, and associated with high patient satisfaction. Most patients indicated they would recommend the procedure.

A small subset expressed partial dissatisfaction, often related to the recurrence of veins or the development of new varicosities. These may represent natural progression rather than treatment failure and are typically suitable for later laser therapy. Patients were informed of this possibility during the consent process.

This study has limitations. Satisfaction scores were self‐reported and thus subject to response bias. Although all patients were examined by the treating vascular surgeon approximately 4–6 weeks post‐procedure, these reviews were recorded as clinical impressions rather than using standardized tools. Future research should incorporate objective assessments, such as clinician‐rated scales or photographic comparisons.

The small sample size and modest response rate limit generalizability. Additionally, formal statistical testing of satisfaction by age or sex was not feasible. No demographic trends were observed, but larger studies would enable more reliable subgroup analysis.

Finally, the retrospective design introduced variability in response timing. Most questionnaires were returned 4–7 years post‐treatment, reflecting long‐term impressions rather than immediate outcomes. Fixed follow‐up intervals would improve future data reliability.

In conclusion, facial phlebectomy under local anesthesia appears to be a safe and effective option for selected patients with prominent facial veins. High satisfaction rates and low complication risks support its continued use in cosmetic practice. Further research with larger cohorts and objective outcome measures is warranted.

## Author Contributions

Conception or design of the work: M.S.W. Data collection: S.D.M. and A.G.N. Data analysis and interpretation: L.D.S. and M.S.W. Drafting the article: L.D.S. and M.S.W. Critical revision of the article: M.S.W. Final approval of the version to be published S.D.M., L.D.S., A.G.N., and M.S.W.

## Ethics Statement

The authors have nothing to report.

## Conflicts of Interest

The authors declare no conflicts of interest.

## Data Availability

The data that support the findings of this study are available from the corresponding author upon reasonable request.
